# Network Structure Implied by Initial Axon Outgrowth in Rodent Cortex: Empirical Measurement and Models

**DOI:** 10.1371/journal.pone.0016113

**Published:** 2011-01-11

**Authors:** Diarmuid J. Cahalane, Barbara Clancy, Marcy A. Kingsbury, Ethan Graf, Olaf Sporns, Barbara L. Finlay

**Affiliations:** 1 Center for Applied Mathematics, Cornell University, Ithaca, New York, United States of America; 2 University of Central Arkansas, Conway, Arkansas, United States of America; 3 University of Arkansas for Medical Sciences, Little Rock, Arkansas, United States of America; 4 Department of Biology, Indiana University, Bloomington, Indiana, United States of America; 5 Biology Department, Amherst College, Amherst, Massachusetts, United States of America; 6 Department of Psychological and Brain Sciences, Programs in Neuroscience and Cognitive Science, Indiana University, Bloomington, Indiana, United States of America; 7 Department of Psychology, Cornell University, Ithaca, New York, United States of America; Newcastle University, United Kingdom

## Abstract

The developmental mechanisms by which the network organization of the adult cortex is established are incompletely understood. Here we report on empirical data on the development of connections in hamster isocortex and use these data to parameterize a network model of early cortical connectivity. Using anterograde tracers at a series of postnatal ages, we investigate the growth of connections in the early cortical sheet and systematically map initial axon extension from sites in anterior (motor), middle (somatosensory) and posterior (visual) cortex. As a general rule, developing axons extend from all sites to cover relatively large portions of the cortical field that include multiple cortical areas. From all sites, outgrowth is anisotropic, covering a greater distance along the medial/lateral axis than along the anterior/posterior axis. These observations are summarized as 2-dimensional probability distributions of axon terminal sites over the cortical sheet. Our network model consists of nodes, representing parcels of cortex, embedded in 2-dimensional space. Network nodes are connected via directed edges, representing axons, drawn according to the empirically derived anisotropic probability distribution. The networks generated are described by a number of graph theoretic measurements including graph efficiency, node betweenness centrality and average shortest path length. To determine if connectional anisotropy helps reduce the total volume occupied by axons, we define and measure a simple metric for the extra volume required by axons crossing. We investigate the impact of different levels of anisotropy on network structure and volume. The empirically observed level of anisotropy suggests a good trade-off between volume reduction and maintenance of both network efficiency and robustness. Future work will test the model's predictions for connectivity in larger cortices to gain insight into how the regulation of axonal outgrowth may have evolved to achieve efficient and economical connectivity in larger brains.

## Introduction

Understanding the nature of the network of interconnections within the cerebral cortex is of central importance to determine how information is distributed and integrated [1]. Collated neuroanatomical data sets from several species [2], [3] on the neuroanatomical connections of multiple cortical regions have been analyzed extensively, examining hierarchical organization, clustered and modular architecture and other key network metrics such as small-world attributes [4]–[6]. More recently, the functional connectivity of the human cortex has been described by analyzing time series of activations obtained in imaging studies, during resting and task-evoked activity [7]. Until now, only a few studies have attempted to trace the developmental origin of key features of cortical network architecture. Understanding the early development of anatomical connectivity is important as it may help to identify the structural features that become organized prior to those which arise in the course of experience.

During the time in which anatomical information has been gathered about the connectional anatomy of the cortex, our computational understanding of it has changed continuously. The classical view of the cortex centered on operations performed by “cortical areas” with each area representing a distinct region thought to integrate specific inputs from thalamus and cortex, transform them, and pass them to “higher” areas for further integration. In accord with this theory, investigations of cortical neuroanatomy and neurophysiology catalogued in great detail patterns of input and output connections, and response properties of single neurons of specific cortical regions, to illuminate each area's essential function (e.g. [8], [9], reviewed in [10]). Correspondingly, early studies of the development of connectivity in the cortex focused on primary visual cortex and primary somatosensory cortex, treating each as independent entities [11], [12]. Few studies were designed explicitly to compare the early establishment of cortical connectivity across areas or to link its local and global features.

Serial-processing or switchboard metaphors for the cortex have been progressively replaced, not least because of the development of functional neuroimaging techniques, by a less hierarchical and more distributed model of function [13]. Under this view single areas may contribute to multiple functions and vice versa [14]–[16]. The assignment of “function” to single brain areas has been shown to be quite plastic, on both short and long timescales [17], [18]. Information gathered from neuroanatomical studies [6], [19] together with advances in graph theoretical analysis of anatomical networks [1] suggest that patterns of cortical connectivity reflect the interplay of local and global rules of how axons become spatially distributed, rather than a fixed developmental program that assigns connections to areas according to a pre-formed list of unique inputs and outputs.

Efficiency and scaleability are key design objectives for networks specialized for information processing, and they also have implications for evolving neural systems [20]. In combination with the efficient transmission of information, neural systems must also be economical in terms of volume and energy consumption, and existing evolved brains are manifestly scalable [21]–[23]. Characterization of the efficiency and scaleability of local and global connectivity patterns in the cortex has been limited by the fact that all of the information presently available for analysis falls into one of two distinct categories, differentiated by the length-scales they examine. The first category, recorded in neuroanatomical studies, by activation of functional areas in imaging studies, or by diffusion tensor and diffusion spectrum imaging, describe axonal projections at scales comparable to the size of the entire cortex but typically at the low resolution of cortical areas or “regions of interest” [24]. The second category of studies has focused on smaller units of cortex, mapping connections within cortical columns or patterns of synaptic connectivity on individual arbors [25].

How features at the large scale emerge from the developmental rules governing growth at the cellular level is not well understood. Anatomical studies of the establishment of connectivity spanning those two length scales are lacking in the literature, as are any attempts to infer the global network structure arising from such wiring rules. For this reason, we undertook to examine the establishment of overall connectivity in the cortex in a small mammal, the hamster, where the cortex is recently formed and axon outgrowth is in progress at the time of birth. Furthermore, we compared the connectivity patterns of small regions across the cortex, both independently of and in relation to their cortical region of origin. Based on our empirical observations, we propose a method of generating model cortical networks. Further, we use the model to make inferences about the particular form of the axon outgrowth distribution observed, arguing that it may be favored because it reduces wiring volume while maintaining high network efficiency and robustness. The ultimate intention is to ascertain, in a small cortex, basic principles for the establishment of axon network structure at the onset of first experience, and examine how those principles scale in expanding cortical sizes.

## Methods

### Data Acquisition and Basic Quantification

#### Ethics Statement

Throughout all experiments, animals were treated in accord with the policies and procedures set forth in The National Institutes of Health Guide for the Care and Use of Laboratory Animals and approved regulations of Cornell University's Institutional Animal Care and Use Committee (IACUC). The experiments described in this paper were conducted under IACUC protocol number 84-55-00.

#### Species

Fifty-four Syrian hamster pups (*Mesocricetus auratus*) of both sexes from timed pregnancies in the laboratory colony were used in this study. Animals were fed *ad libitum* and maintained on a 10L:14D photoperiod.

#### Tracer and injections

According to convention, the 24-hour period following birth is designated postnatal day 0 (P0). Only hamsters born within 24 hours of the expected 15.5 day gestation period were used for this study. Biocytin was injected into pup cortex at ages P0, P2, P4, P6 and P8, with transport time optimized at 24 hours. Intracortical transport was principally anterograde with very few cortical cell bodies retrogradely labeled outside the immediate injection area. However, both anterograde and retrograde transport were observed to the thalamus, although at ages earlier than P4, transport to the thalamus was principally retrograde. This thalamic label was used to identify thalamic nuclei with connections to the cortical injection site.

#### Surgeries

Pups were anesthetized by hypothermia and maintained on an ice blanket in molded head and body restraints. The skull was exposed and a hole made overlying the cortical region of interest. A solution of 5% biocytin was injected through a backfilled micropipette (inner diameter 15–20 µm) using a Picospritzer (General Valve Co.; Fairfield, NJ), with pressure and duration adjusted to deliver >0.1–0.5 microliters of solution. Injections were positioned only in cortical regions that could be clearly viewed, avoiding areas of high vascularization. Because rodent intracortical connectivity originates from both infra- and supragranular layers, injections were centered at a depth adjusted for the different ages to span the full thickness of the cortex while avoiding the underlying white matter. Following injections, the scalp was sutured; pups were rewarmed and returned to the mother. After 24 hours pups were overdosed with sodium pentobarbital and perfused transcardially with 0.9% saline followed by 4% paraformaldehyde and 0.1% gluteraldehyde in 0.1 M phosphate buffer (PB, pH 7.4). Brains were cryoprotected in 30% sucrose at 4° centigrade until processing.

#### Histochemistry

All brains were frozen and sectioned coronally at approximately 60 µm. Sections were treated according to a protocol adapted from Ding and Elberger [26], followed by conventional diaminobenzidine (DAB) processing. Briefly, sections were rinsed in phosphate buffered saline (PBS; pH 7.2,) quenched in 1% H_2_O_2_, and immersed in 1% Triton-X100 (TX) in PBS. Tissue was incubated overnight in an avidin-biotin solution (1∶100; Vectastain Elite Standard Kit) containing 1% TX. Sections were reacted with 0.004% tetramethylbenzidine (TMB); mounted on chromium-gelatin coated slides, dehydrated, cleared, and coverslipped with Krystalon (Fisher Scientific) with one series counterstained with cresyl violet. Dehydration of the unstained series was kept to less than one-minute immersion in each of three graded alcohols to minimize shrinkage. Following data collection, this series was also lightly stained with cresyl violet to further verify neural divisions and landmarks.

#### Reconstruction of dorsal cortex and injection sites

Reconstructions were made using a LeitzDiaplan Microscope and a Neurolucida imaging system with a mechanical stage (Microbrightfield, Inc., Colchester, VT). Measurements were obtained from each traced serial section in each of the fifty-four brains, always including sections containing landmarks comparatively stable across development such as the furthest ventral and caudal levels of the white matter, thalamic complex, and caudate nucleus. To avoid artificially elongating in the medial to lateral plane when converting from coronal sections to dorsal views, in each traced section a midpoint contour was measured using a line drawn intermediate between the superficial white matter and the top of cortical layer I. The measurement began medially at the “point of flexure” (dorsalmedial crest separating the two cerebral hemispheres) and extended laterally to the rhinal fissure. A dorsal cortical surface view was then constructed by plotting each midline measurement to scale using Canvas 6.0 (Deneba Systems, Inc.). In effect, this method generates a flattened or “unrolled” surface from curved serial coronal sections as if viewed from above (the dorsal surface; see Figure 4D).

#### Identification of cortical regions

Multiple sources of information were integrated to position general areal boundaries in the developing cortex and locate injection sites. First, atlases of the adult hamster brain [27] and developing and adult rat brain [28]–[30] were used to establish the overall orientation and form of the map. It is well established that the topology of thalamocortical and corticothalamic projections is conserved from first innervation to adulthood, though maturational gradients in the cortex and the deformation of the cortex by overall growth alters the relative size of cortical regions [31]–[34]. The anterograde and/or retrograde transport of biocytin from injection sites to the thalamus, listed in Table 1, was used to fix the positions of the primary visual, auditory and somatosensory cortex and shift areal boundaries with respect to the adult cortex as required, for each postnatal age. Published and unpublished hamster developmental studies from this laboratory were also used to help align regions of the developing cortex with respect to other adjoining telencephalic regions, such as the striatum and hippocampus, whose topological positions remain fixed from initial generation to adulthood [33], [35], [36].

**Table 1:** Injections of anterograde tracer were made into three cortical regions: anterior (presumptive motor), middle (presumptive somatosensory) and posterior (presumptive visual cortex).

	Age	Animal	Pup weight	A-P Length	Label	Placement
1	PO-1	704.4	2.9 g	3420μm	VL, VB, PoM	anterior
2	PO-1	697.1	2.4 g	3540 µm	VL, VB	anterior
3	PO-1	675.2	2.8 g	3600 µm	MD, VL, VB	anterior
4	PO-1	678.3	2.6 g	3660 µm	VL, VB, R	middle
5	PO-1	704.2	2.7 g	3720 µm	VL, VB, R	anterior
6	PO-1	678.1	2.6 g	3600 µm	MD, VL, VB	anterior
7	PO-1	678.2	2.8 g	3720 µm	VL, VB, dLGN	posterior
8	PO-1	675.1	2.7 g	3480 µm	R, L	posterior
9	PO-1	678.4	2.6 g	3600 µm	VB, L, dLGN	posterior
10	P2-3	674.3	2.6 g	3300 µm	VB	anterior
11	P2-3	695.1	2.8 g	3900 µm	VB	anterior
12	P2-3	695.3	2.8 g	3420 µm	VL, VB, L	anterior
13	P2-3	710.1	4.4 g	3720 µm	VL, VB, L	middle
14	P2-3	695.2	2.8 g	3540 µm	VL, VB, R	middle
15	P2-3	679.1	3.6 g	4020 µm	dLGN, vLGN	posterior
16	P2-3	710.4	4.0 g	4020 µm	dLG	posterior
17	P4-5	671.4	4.4 g	4560 µm	VL, VB, R	anterior
18	P4-5	680.4	4.2 g	4140 µm	VL, VB, R	anterior
19	P4-5	681.2	4.0 g	4200 µm	-	middle
20	P4-5	694.3	4.2 g	4140 µm	R	middle
21	P4-5	697.3	6.8 g	4080 µm	PoM, L, dLGN, vLGN	posterior
22	P4-5	694.2	4.2 g	4140 µm	VL, R	posterior
23	P6-7	680.5	7.4 g	4500 µm	VB	anterior
24	P6-7	669.5	6.2 g	4980 µm	VL, VB, R	anterior
25	P6-7	694.4	7.8 g	4720 µm	VL, VB, PoM, R	middle
26	P6-7	683.1	6.4 g	5040 µm	VL, VB, L	middle
27	P6-7	707.1	5.1 g	4620 µm	VL, VB, R, L	middle
28	P6-7	683.3	7.2 g	5040 µm	L, dLGN, vLGN	posterior
29	P6-7	669.4	6.5 g	4800 µm	L, dLGN, vLGN	posterior
30	P6-7	708.2	7.4 g	5340 µm	R, L, dLGN, vLGN	posterior
31	P8-9	671.6	9.1 g	4920 µm	VL, VM	anterior
32	P8-9	672.8	10.8 g	5100 µm	VL, VM, PoM	anterior
33	P8-9	679.7	10.8 g	5100 µm	VL	middle
34	P8-9	692.4	7.9 g	4380 µm	VL, VB, R, L, dLGN, vLGN	middle
35	P8-9	701.3	10.0 g	4980 µm	L, dLGN, vLGN	posterior
36	P8-9	701.1	9.2 g	5160 µm	R, L, dLGN, vLGN	posterior

Because some variability is evident in the A/P length of brains at similar early ages, we also list pup weight in grams. The Label lists only those putative major thalamic nuclei in which we have a great degree of confidence in identification at these ages; other nuclei were also labeled (see also [56]).

Abbreviations: dorsal lateral geniculate nucleus, dLGN; lateral nucleus, L; mediodorsal nucleus, MD; posteromedial nucleus, PoM; reticular nucleus, R; ventrobasal nucleus, VB; ventrolateral nucleus, VL; ventral lateral geniculate nucleus, vLGN.

#### Axon tracing

Thirty-six developing brains with well-labeled axons were completely analyzed microscopically and 24 representative brains were traced using a Neurolucida (25×) for more detailed morphological and statistical analysis, including ages (injected-recovered) P0–1 (n = 7), P2–3 (n = 4), P4–5 (n = 3), P6–7 (n = 4), P8–9 (n = 3). Axons were identified by their coloring, thin uniform appearance, characteristic branching patterns, and, on many occasions, the presence of growth cones. Every visible intracortical axon in each traced section was drawn. Sections to be traced (typically over one half) were determined by the presence or absence of labeled axons, although as noted above, sections containing the furthest ventral and caudal levels of the white matter, thalamic complex, and caudate were always traced to obtain registration measurements for dorsal views.

#### Reconstruction of axonal projections

The tracings of coronal sections were then used to generate dorsal view reconstructions of the furthest distal points where labeled axons were found, as well as axon density plots of projections arising from injection sites. First, radial lines were drawn perpendicular to the middle layers of the gray matter and spanning the entire depth of the white and gray matter, spaced every 200 µm beginning at the point of flexure and ending at the rhinal fissure, with the last measurement the interval between the final 200 µm line and the rhinal fissure. Tangential substrates were then outlined using pseudo phase-contrast on unstained tissue and adjusted using counterstained sections (accounting for shrinkage, which was consistently less than 10%). The tangential substrate boundaries included the subjacent border of the cortex (layer VI), the subjacent border of the infracortical fasciculus (a cell-sparse area above the subplate neurons, also called “channel 2” in [29], and the subjacent border of the subplate neurons, which corresponds to the superficial border of the white matter. Axons crossing each radial line were counted and tangential substrate subtotals obtained. Each of these counts was associated with a location (*i,j*) on the 60 µm×200 µm grid, indicating that this is the count at the *i*th radial line in the *j*th section. The substrate subtotals were labeled 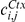, for those axons counted in cortical layers, and 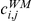, for those in the white matter. We use 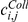 to denote the collapsed count of all axons encountered at a radial line, i.e. for each count location 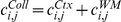.

The tangential compartments are not uniformly identifiable in hamster cortex: in far anterior and posterior coronal sections, white matter fibers, subplate neurons and the infracortical fasciculus merge. Moving anterior to posterior in pup brains, white matter fibers are first noted at the level of orbital cortex where the rhinal fissure no longer clearly separates cortex and olfactory bulbs, followed approximately 0.5 mm posterior by a distinct layer of subplate neurons and approximately 0.5 mm further posterior by the band of fibers comprising the infracortical fasciculus. In far lateral regions of cortex, the subplate neurons seem to merge with neurons of the claustrum; in both far lateral and posterior cortex, the fasciculus is quite thick relative to anterior sections (see also [37]). The position of isolated single axons or very sparse projections was always registered independent of the radial lines used for systematic sampling. Care was taken to ensure that counts did not include axons traveling to subcortical or callosal areas; however, although these axons travel different routes, some small uncertainty is unavoidable. Dorsal view reconstructions were produced for projections traveling from an injection site in each of the different substrates (cortical layers I–VI, infracortical fasciculus, subplate and white matter), as well as a “collapsed” view of the first three combined so as to represent the conventionally identified cortical gray matter.

#### Basic quantification

The surface area covered by underlying axons within the borders of the cortex bounded by the point of flexure and rhinal fissure was determined for 20 traced pup brains using NIH Image. Total area of axon coverage (in mm^2^) was analyzed for cortex, subplate, infracortical fasciculus, and for a category collapsed across these three, as well as for the white matter. These totals were expressed as a percentage of the total dorsal area or anterior/posterior (A/P) or medial/lateral (M/L) length in each individual brain. Schematized cortical areas were not used in statistical analysis; areas were determined for each individual brain at each age (see also Table 2). ANOVA was performed in Statview 5.0 to determine if axon extent in M/L or A/P planes varied based on injection site location or age, followed by Scheffe's Post-hoc test when appropriate.

**Table 2:** Statistical analysis was performed using these measurements from 20 pup brains and 2 adult brains.

	Age	Animal	Placement	Total Area	WM	Cortex	Subplate	Fasc.
1	P0-1	697.1	anterior	17.4 mm^2^	56.1%	60.1%	39.0%	35.7%
2	P0-1	675.2	anterior	15.0 mm^2^	23.1%	50.8%	20.9%	20.0%
3	P0-1	704.2	anterior	16.0 mm^2^	25.1%	36.1%	12.1%	16.9%
4	P0-1	678.1	anterior	15.6 mm^2^	58.3%	62.6%	50.4%	53.9%
5	P0-1	678.2	posterior	18.1 mm^2^	21.2%	38.1%	19.5%	33.2%
6	P0-1	675.1	posterior	16.4 mm^2^	50.2%	73.6%	49.6%	56.7%
7	P0-1	678.4	posterior	17.5 mm^2^	23.4%	55.7%	31.9%	35.0%
8	P2-3	695.3	anterior	18.3 mm^2^	48.2%	66.1%	30.8%	37.1%
9	P2-3	710.1	middle	16.7 mm^2^	67.0%	79.4%	32.8%	33.5%
10	P2-3	695.2	middle	19.2 mm^2^	49.4%	88.2%	36.6%	46.4%
11	P2-3	679.1	posterior	25.0 mm^2^	35.4%	59.0%	39.4%	22.8%
12	P4-5	671.4	anterior	30.8 mm^2^	32.0%	59.1%	23.1%	17.9%
13	P4-5	680.4	middle	31.4 mm^2^	20.5%	53.8%	16.1%	16.9%
14	P4-5	697.3	posterior	28.6 mm^2^	35.7%	40.6%	31.0%	10.8%
15	P6-7	680.5	anterior	35.9 mm^2^	32.1%	50.9%	29.2%	22.6%
16	P6-7	669.5	anterior	40.2 mm^2^	25.6%	71.3%	22.7%	24.9%
17	P6-7	683.3	posterior	44.6 mm^2^	48.7%	52.2%	43.3%	29.1%
18	P8-9	672.8	anterior	39.6 mm^2^	46.3%	81.2%	47.1%	48.0%
19	P8-9	671.6	anterior	37.2 mm^2^	22.8%	73.4%	23.7%	14.8%
20	P8-9	701.3	posterior	35.7 mm^2^	40.9%	71.8%	47.5%	33.6%

Total isocortical area is expressed in mm^2^ and axonal coverage in each substrate is expresses as a fraction of total isocortical area. Abbreviation: white matter, WM; infracortical fasciculus, fasc.

### Characterization of Axon Outgrowth Distribution

#### Interpolating the data

The data for each animal was recorded as a set of axon counts taken at points on a 2-D grid whose axes aligned with the medial/lateral (ML) and anterior/posterior (AP) axes of the flattened cortical hemisphere. Indexing each grid point (*i,j*) and calling the corresponding count 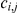, these counts document the number of axons originating at the injection site which were detected at location (*i,j*) on the 200 µm by 60 µm grid of sample points. As described above, for each animal, three sets of count data were analyzed: axons found in the cortical layers only 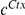, axons in the white matter only 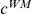, and a collapsed set 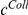, where 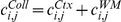.

In order to arrive at the desired description in terms of probability distribution functions of axon terminal sites, the following steps were carried out. Any missing counts from the interior of each dataset were interpolated. Each grid was re-centered such that the injection site (detected as the site having the maximum axon count 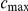) had index 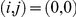. We assumed that the injection site is a point source of axons. To simplify further calculations, a 2-dimensional first order interpolating function was fitted to each grid (using Mathematica). With the interpolating function 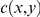, it was possible to treat the count data as continuous over the 2-D domain with 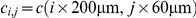.

#### Angular distribution of outgrowing axons

**Figure 1:**
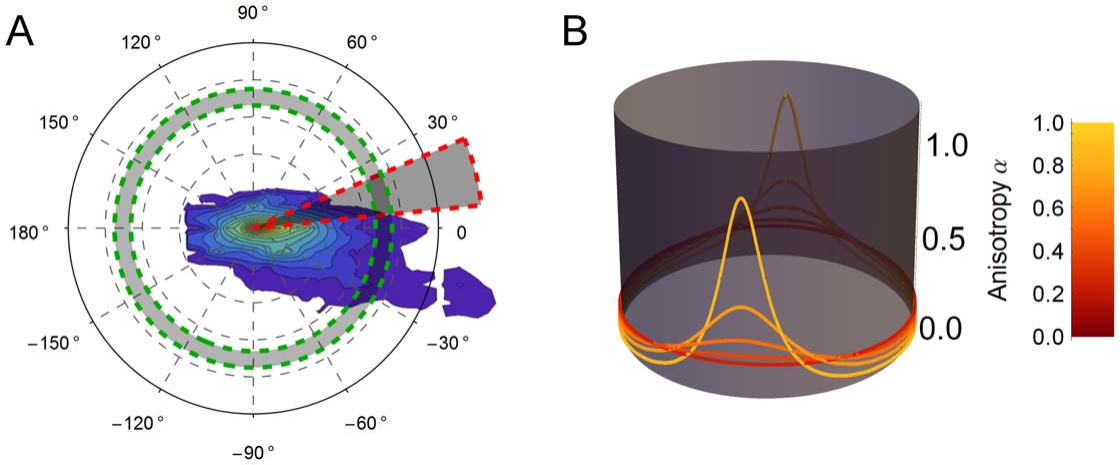
Characterizing axon outgrowth distributions. (**A**) The contour plot depicts a typical distribution of axon outgrowth from the site at the origin. The spatial distribution of axon outgrowth was described by an angular distribution function *υ*(*θ*), quantifying the relative fraction of outgrowth volume in the infinitesimal sector of the plane centered at angle *θ* (shaded sector). A radial distribution function *S*(r) quantifies the fraction of axon volume in the infinitesimal annulus at radius *r*. (**B**) The function *q*(*θ;α,η*), with parameter *α* determining the height of its two diametrically opposed peaks, is graphed here on a circular domain for several different values of *α*. The tilt *η* is the same for each member of the family shown here, resulting in the peaks occurring at the same values of *α* and *α +* 2*π* for each member. The distribution is almost uniform on the circle for values of *α* near zero. As *α* approaches 1, then *q*(*θ;α,η*) becomes a delta function.

Two functions, calculated using 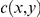, were used to characterize each dataset. The first, 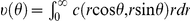, accounts for the angular distribution of axons (see Figure 1A). The data exhibited a prevalence of growth along the direction of the ML axis in preference to the AP axis. To quantify this anisotropy, we fitted a double peaked function 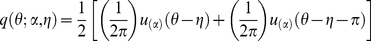,defined on 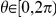. Here 
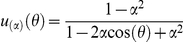
 with parameter 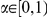 is a probability distribution on the circle (see Figure 1B). The distribution is flat at 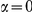 and approaches two delta spikes as 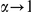. For each animal, we calculated the values of 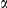 and 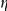, or the *anisotropy* and *tilt* as we call them respectively, which minimized the least squares error between 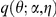 and 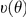 (see Figure S2 for fits to data). Thus 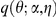 is a probability distribution for the relative volume of outgrowth in each direction, parametrized in each case by anisotropy 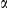
and tilt 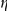.

#### Radial distribution of outgrowing axons

Arriving at the radial distribution function, characterizing the length distribution of the axons, requires taking into account the cumulative nature of the count data: 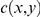 is the number of axons one would expect to find passing through any point 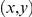, not the number of axons terminating there. Given that axons may trace more or less circuitous routes between their origin and terminal arbor, and may also branch en route, it will not be possible to exactly recover the density distribution of endpoints from the count data. To arrive at an approximation to the true distribution, we assume (i) that no branching occurs prior to arrival at the terminal site and (ii) that axons travel from the origin along straight trajectories. These assumptions are generally consistent with the data collected in this study and in earlier work [33]. Given that our network model is constructed using such straight-line axon trajectories, disregarding the (unknown) particulars of axons routes will not affect our simulated networks.

The quantity 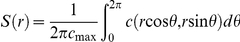, under assumptions (i) and (ii) above, can be interpreted as the probability that an axon has length greater than or equal to *r* (see Figure 1A). The probability that it terminated at a length less than *r* is simply 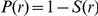 and so, in principle, 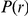 is an approximation to the cumulative distribution function (CDF) for the length of the axons. In practice however, portions of the empirical “CDFs” fail to meet the monotonicity property required of distribution functions. The difficulty arises near the origin, at small radii *r*, where the resolution of the experiment means that few count sites are contributing to the calculated value of 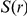. Furthermore, what counts are present may be noisy due to the high concentration of stained axons close to the injection site. For this reason, we chose to disregard the non-monotonic portions of the empirical functions 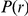
near the origin and fit analytical CDF's to the remaining monotonic data. However, it is that portion of the data, near the origin, which would otherwise provide the normalizing constant for our distributions (i.e. 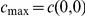). Hence, the fitting procedure must also provide an estimate for that normalizing constant. On inspection, the usable portion of the empirical distributions seemed to be well fit by gamma distributions, which have previously be used to model axon length distributions [38]. So as to reduce the number of fitting parameters, we constrained the fitting procedure to use gamma distributions with a shape parameter equal to 2. Thus the radial distribution of each outgrowth pattern was characterized by the mean 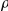 of its fitted gamma distribution, 
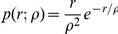
. See Figure S1 for fits to data.

### Network Modeling

#### Generating spatial networks

**Figure 2:**
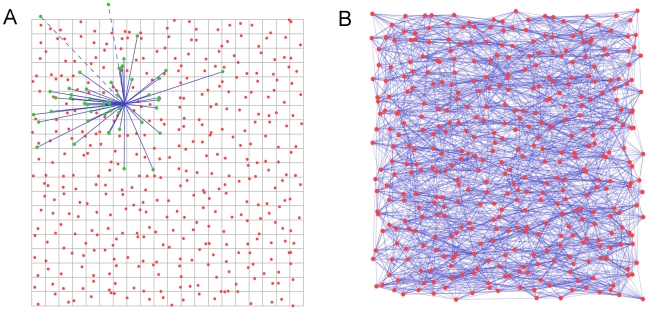
Generating spatial networks. (**A**) We begin with a grid of 100µm squares, each containing a node located uniformly at random within the square. For each node, a set of candidate end points for efferent links is drawn from under the probability distribution, those terminating outside the boundary of the region (such as the dashed lines here) will be discarded in favor of new candidates until the quota of *n_axons_* has been reached. The ends of the candidate axons snap to the nearest node, and the links are recorded in the adjacency matrix. (**B**) Repeating the procedure in (A) for each node results in a network such as that depicted here. For clarity of display, the network in (B) is drawn on a grid of 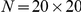 nodes having *n_axons_  = * 10. Networks used in our simulation have 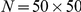 and *n_axons_  = * 10.

We study a network model whose nodes are localized populations of neurons, linked by edges which model representative axons. The network is constructed as follows (see also Figure 2). We model the hamster's cortical hemisphere as comprising 2,500 idealized cortical units. Each unit represents the neural population under a 100 µm×100 µm square of cortex. Our model cortex is a grid of 50×50 = 2,500 of such units, thus mimicking a cortical sheet measuring 5 mm×5 mm. These units are the nodes in our network. With each node *i* we associate a location 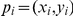, chosen uniformly at random within the corresponding unit's square footprint.

Emanating from each node are a fixed number, 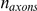, of directed network edges, representing efferent axons. Edges are assigned to each node *i* by using the following procedure: *(i)* select a length *r* and a direction 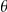 from under the empirically derived radial and angular probability distributions, 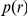 and 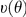, respectively; *(ii)* find the node *j* nearest to the point at distance *r* from node *i* in the direction 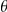; *(iii)* add a directed edge, pointing from node *i* to node *j*, to our network. There are two exceptions to the above procedure. First, if the randomly chosen length *r* and direction 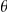 determine a point which lies outside the grid, we chose a new random *r* and 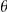, repeating until the chosen *r* and 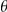 determine a point inside the grid. Second, self edges are disallowed – should one occur, we discard it and choose a new random endpoint such that the axon does not terminate at its node of origin. The procedure for generating edges is repeated until the node *i* has been assigned its full complement of 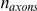 edges. Edges are assigned to all nodes in the network in this manner. Once all edges have been assigned, in the *adjacency matrix A*, the entry *A_ij_* records the number of directed edges from node *i* to node *j*.

We note that although the details of meandering axonal paths were ignored as we deduced 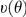 and 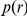, our method of generating networks does not require such details to reproduce the distribution of axon terminal sites. Our networks were created and visualized using Mathematica 7.0 [39].

### Analysis of Networks

#### Degree distribution, path lengths and clustering

We calculated several measures to analyze the networks generated by our spatial model. The *out-degree* 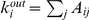 of a node on a directed network counts the number of edges originating at that node. By construction, we have 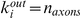 for all nodes in our networks. The *in-degree* counts all edges terminating at a node: 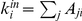. The number of edges comprising shortest network path between any pair of nodes, *i* and *j*, also known as the *distance* from *i* to *j*, is recoded as 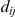. The *average shortest path*, 
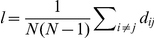
, gives a characteristic length for the network paths. The *clustering* of a network gives the probability that if the edges *i→j* and *j→k* are both present then so too is the edge *k→i*. The clustering, *C*, is given by 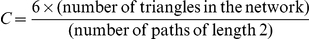, where, for example, *i→j→k* is a path of length 2 and a “triangle” refers to a case where the three edges (*i→j*, *j→k* and *k→i*) are present [40]. A *small-world network* is characterized by having both the short path lengths typical of a random network and the high clustering typical of a more regularly wired (e.g. lattice) network. Using random networks as a baseline, the *small-world index*, *S*, makes the classification of networks as being small-world quantitative [41]. The small-world index of a network with *N* nodes, *M* edges, clustering *C* and average shortest path length *l*, is given by 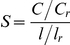, where 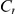 and 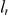 are, respectively, the clustering and average shortest path length of a random network also having *N* nodes and *M* edges. For a random network, 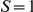, but *S* takes larger values for small-world networks.

#### Network efficiency

*Global network efficiency* quantifies the efficiency of communication between all pairs of nodes on the network, under the assumption that information flows along the shortest paths available [42]. Considering just one pair of nodes first, if an edge joins the two nodes, the path between them has length 1 and so communication is maximally efficient for that pair: we say that path has an efficiency of 1. If the shortest path between a pair of nodes (*i* and *j*) has length 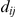, then we say its efficiency is 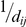. The average value of that pairwise efficiency, taken over all pairs of nodes in the network, is the global efficiency: 
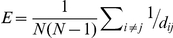
. Only a completely connected network (where all possible edges are present) has a global efficiency of 1. All other networks have an efficiency 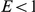.

#### Node centrality

The *betweenness centrality* of a node is a measure of how important that node is for efficient communication on the network [40]. Considering the set of all shortest paths on the network, we see that some “central” nodes may feature in a greater number of shortest paths than do other less central nodes. The betweenness centrality of a node *i* is the fraction of all shortest paths on the network which pass through *i*. Specifically, if 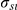 is the number of shortest paths from *s* to *t*, and 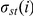 is the number of such paths containing the node *i*, the betweenness centrality of *i* is given by 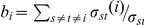.

#### Modularity

It can be useful to think of the nodes on a network as being members of different communities. To investigate the modular nature of our networks, we will assign nodes to non-overlapping communities whose membership is defined by location. If the communities are chosen well, one should observe a greater prevalence of intra-community edges over inter-community edges than would be found in a comparable random network (i.e. a randomly wired network with the same number of nodes and edges). The *modularity Q* quantifies the extent to which we have such a prevalence [43]. Assigning each node to a community, and denoting the node *i*'s community *c_i_*, we can measure the modularity of the network with respect to that community assignment:



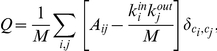
where *M* is the total number of edges in the network and 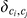
is equal to 1 if 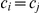 and is zero otherwise. We measure the modularity of our networks with respect to two different, spatially defined, community assignments. First, we partition the nodes into 4 rectangular communities, roughly equal in size, aligned with the medial-lateral axis. Namely, the communities contain the nodes in rows 1 through 13, 14 through 25, 26 through 37 and 38 through 50 of our 50×50 grid, respectively). The communities are thus rectangles whose width spans the medial-lateral dimension of our gird and whose height is about one fourth that of the grid. Second, we use a similar partitioning, but in this case assigning nodes by column number to one of four rectangular communities aligned with the anterior-posterior axis.

The NetworkX package [44] for Python was used to carry out the graph analysis of our networks.

#### Spatial modeling of links

**Figure 3:**
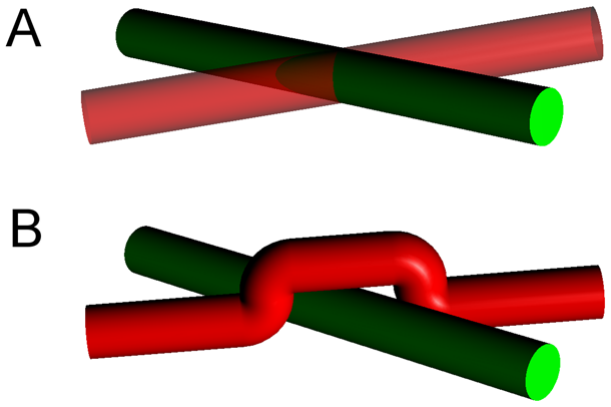
Extra volume cost due to axon encounters. Axons, whose paths were destined to intersect in (**A**) incur an extra volume cost as one or both alter their paths to avoid collision such as in (**B**). The extra volume requirement of scenario (**B**) as compared with (**A**) is 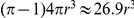, where *r* is the axon radius.

Exploiting the spatially embedded nature of our network, we investigate how anisotropy may affect the volume requirement of axons via an altered number of axon encounters (see Figure 3). Modeling all the axons as straight lines in the plane, we count the number of axon encounters. Only encounters along the body of an axon are considered, those occurring at a node are disregarded. Axon encounters were enumerated using an algorithm implemented in c++.

## Results

### Characterization of initial axon distribution in empirical data

**Figure 4:**
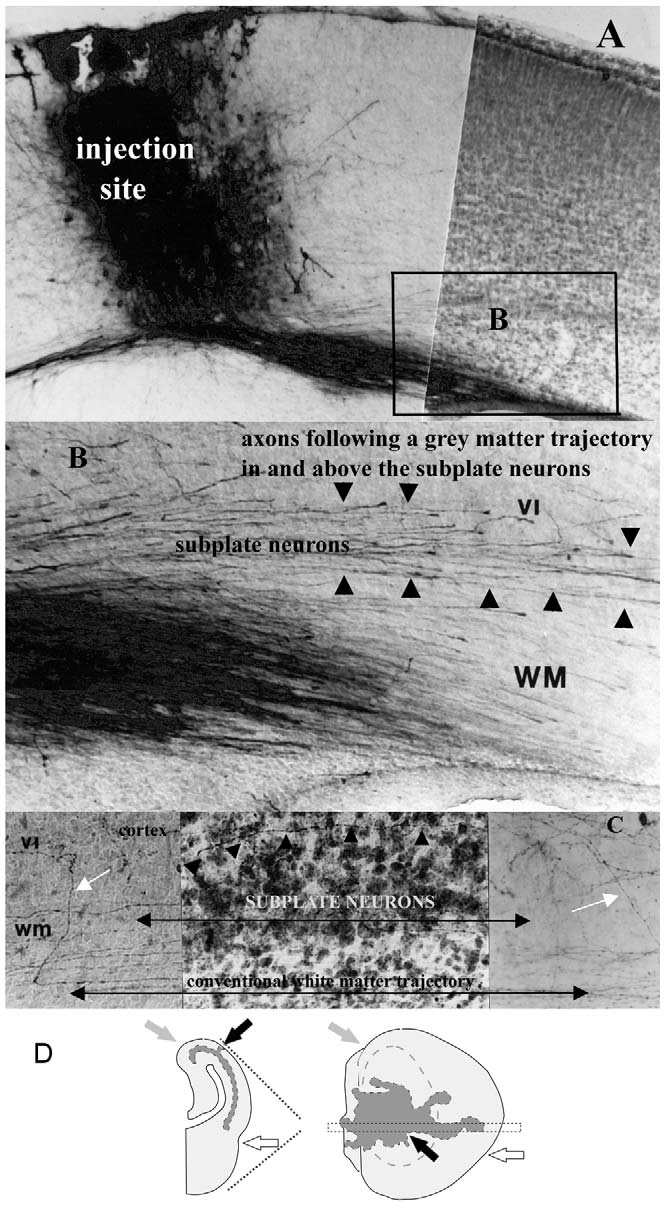
Representative injection site, axon travel in tangential substrates and initial reconstruction procedure. (**A**) Photomicrograph of a biocytin injection site spanning the cortical layers and avoiding the white matter at age P4–5 (injected-recovered). Note that this figure depicts a relatively large injection site, shown here to best illustrate axons traveling in the various tangential substrates. (**B**) High power photomicrograph of area outlined in (A) depicting axons coursing through the cortex, the infracortical fasciculus, and the subplate, as well as in the conventionally identified white matter. (**C**) Although some axons travel within the subplate neurons (visible in the Nissl-stained middle panel of the photomicrograph), others clearly avoid this substrate (small black arrows). Arrowheads in B and C delineate the upper boundaries of the infracortical fasciculus (top), the subplate neurons (middle) and the conventionally identified white matter (bottom). Scale bars are approximately 100 µm. (**D**) Graphic depicting the method by which coronal tracings of axons are mapped onto unrolled dorsal views of the cortex, with the standard dorsal view superimposed in gray on the unrolled cortex. Unrolled cortex includes cortical areas ventromedial and ventrolateral to flexures and thus hidden in a standard (“rolled”) dorsal view. Gray arrows indicate the medial point of flexure in both views; open arrows point to the rhinal fissure. Black arrows in both (A) and (B) indicate the site of the injection in a P0–1 animal (#675.1). Images in this figure were produced using digitally scanned negatives (1200 pixels per inch resolution on a flatbed scanner) processed using PhotoShop software (Adobe, Mountain View, California) to optimal contrast and sharpness, then cropped and lettered. No other adjustments were made.

Thirty-six developing brains were judged to have well-placed injections and well-labeled axons and form the empirical corpus on which the network modeling results are based. Injections were placed across the cortical field, although because of its small size, inaccessibility of the most lateral aspect, developing vascularization, and the relative immaturity of posterior regions at the earliest ages a uniform grid of sites is difficult to produce. Our method of representing initial transected axon counts is shown on a representative “unrolled” P0–1 cortex in Figure 4D.

For initial contrasts of differences in axon outgrowth patterns across the cortical surface, injection placement was assigned to one of three broad categories: “anterior” (presumptive motor), “middle” (presumptive somatosensory) and “posterior” (presumptive visual) cortex (Table 1). The assigned divisions take into account the location of anterograde and/or retrograde labeling found in thalamic regions as well as the position of the axons in the developing cortex. Tracer was typically deposited at each site throughout the layers of the cortex, avoiding the white matter; injections sites were very small compared to the dimensions of the primary cortical areas (Figure 4). The topography of axon extension in each tangential substrate, topography of extension within the cortex as a function of injection site and age, variations in trajectory patterns, and changes in the relative density of projections across ages were all quantified and contrasted.

#### Paths of axon extension

The greatest numbers of intracortically confined projections are local, extending in a radial fashion for short distances in the gray matter directly adjacent to the injection site. Longer-range projections traveling away from injection sites take multiple paths, coursing through the conventionally identified gray matter, the infracortical fasciculus, and among the subplate neurons, as well as in the white matter itself (see Figure 4B). In these small brains, axons reaching the most distant point from the injection site might equally be found traveling through the cortex or through subcortical white matter — the route taken by an axon, within grey matter or white matter did not dictate the distance traversed. Although this study traces the distribution of a population of labeled axons, the trajectories of individual axons were noted when they could be followed. Some of the individual axons that could be traced from injection site to growth cone or terminal arbor traveled exclusively in one substrate, others switched pathways, for example, from the infracortical fasciculus to the white matter, avoiding the subplate (Figure 4 C); or alternated travel between two substrates.

#### Overall axon extension by region, lamina and postnatal day

**Figure 5:**
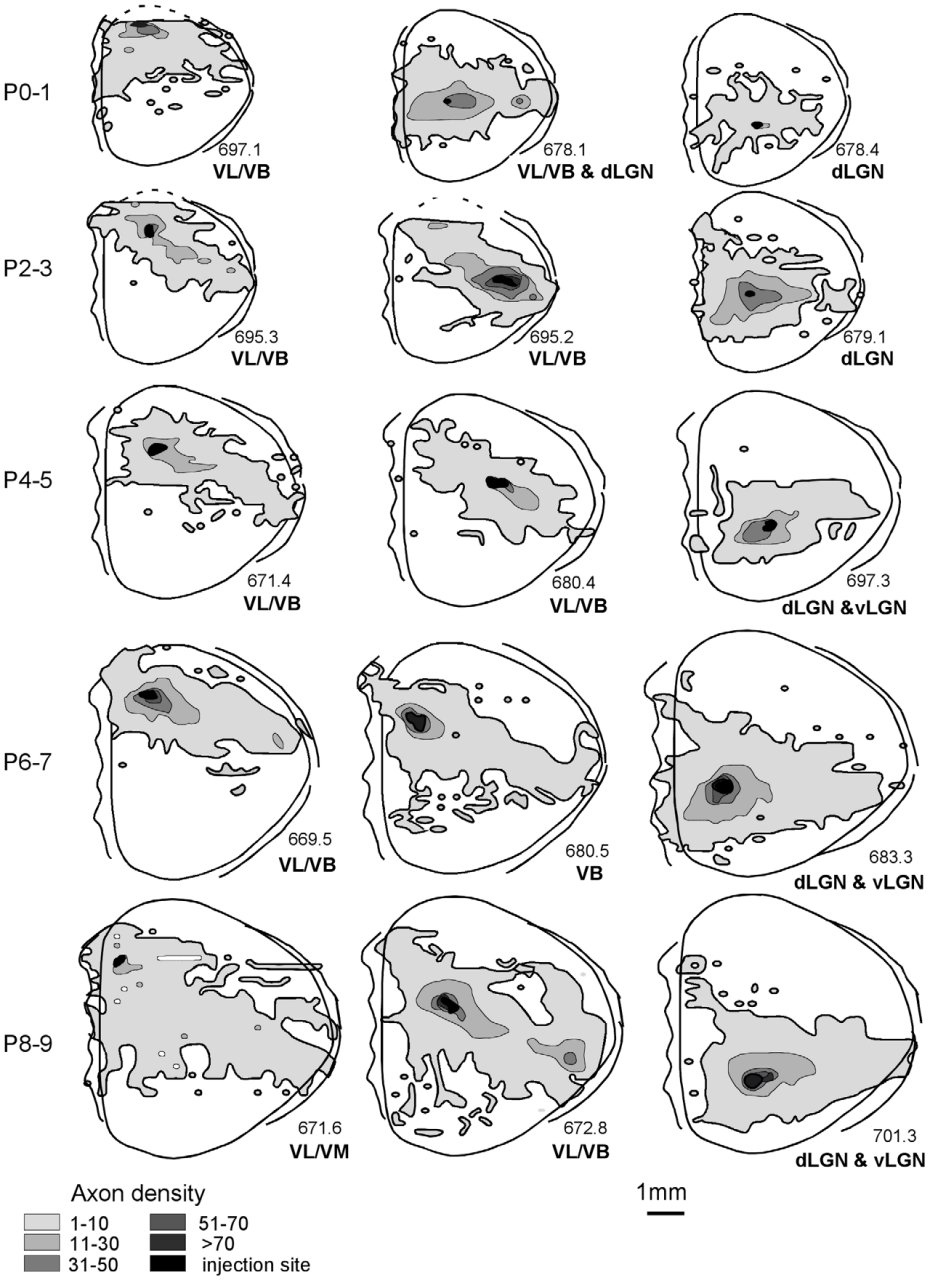
Axon extension in gray matter across early ages. Dorsal views depict representative axon extension in 15 cortices across the different developmental ages included in this study. For this figure, the three tangential substrates that make up the conventional rodent gray matter (subplate, infracortical fasciculus, cortex) are collapsed into one compartment. The abbreviations below each animal indicate the thalamic areas in which anterograde and/or retrograde labeling was noted. Abbreviations: dorsal lateral geniculate nucleus, dLGN; ventrobasal nucleus, VB; ventrolateral nucleus, VL.

**Figure 6:**
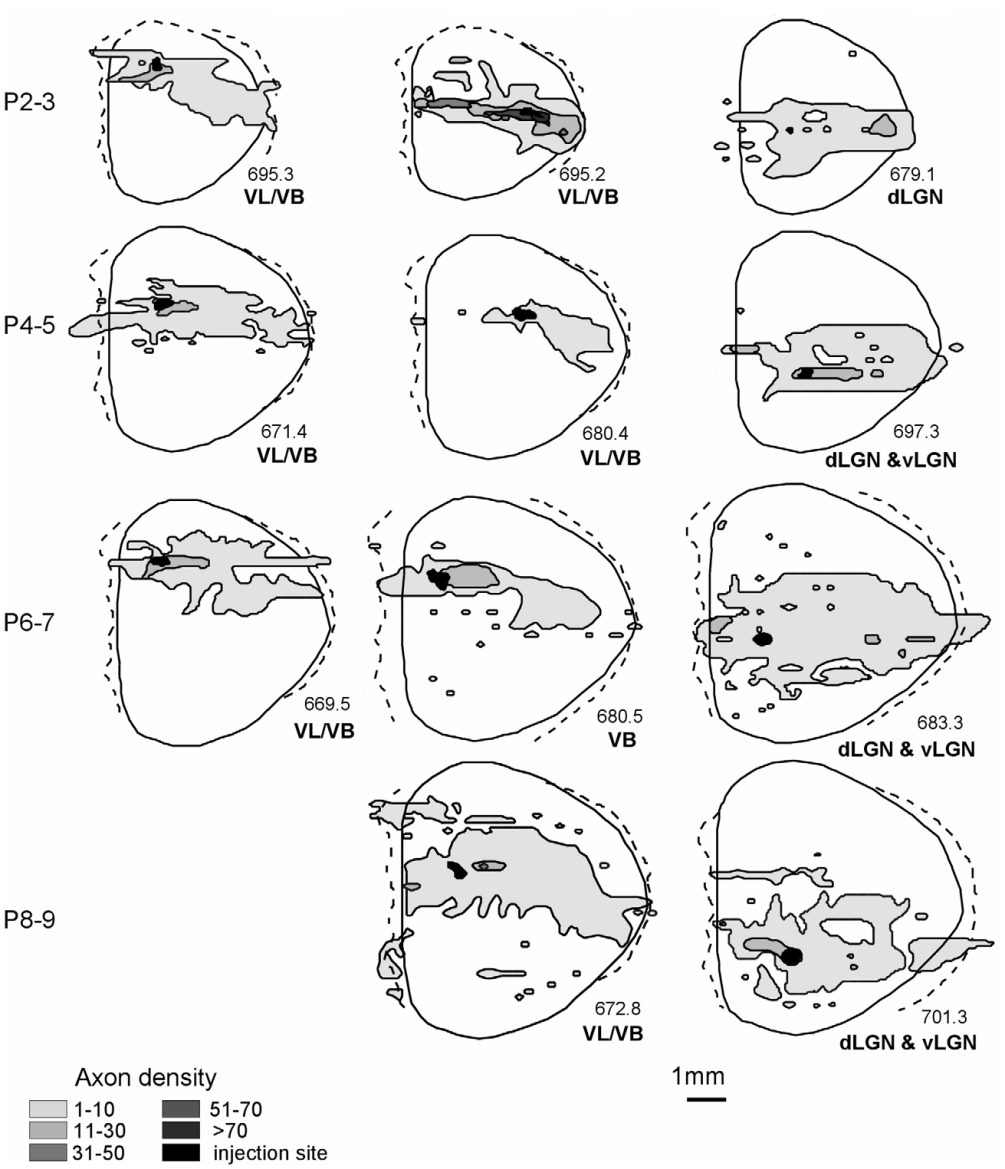
Axon extension in white matter. Dorsal views depicting representative axon extension in the white matter of the same animals depicted in Figure 5.

The mean of the available neural area in each substrate covered from the injections (expressed as a percentage of total dorsal cortical area) is as follows: cortex mean: 61.21%, SE: 3.24, white matter mean: 38.09%, SE: 3.16; subplate mean: 32.34%, SE: 2.59; infracortical fasciculus mean: 30.53%, SE: 2.97 (Figures 5,6). Thus, these small tracer injections resulted in extensive axon spread in the cortex, and insofar as it was possible to rank injection sites by size, the only difference associated with injection size was projection density, not extent.

Even at the earlier age, connections from each site span almost the entire medial/lateral (M/L) distance of the cortex; this coverage persists as total cortical area more than doubles between ages P0–1 and P8–9 (Table 2). This widespread axon extension, evident across all locations, is illustrated in the dorsal reconstructions depicted in Figure 5. Because extension within the subplate and infracortical fasciculus was always less than extension within the cortex itself, in Figure 5 these three divisions are collapsed into one “gray matter” compartment. Qualitative inspection and statistical analyses indicate no significant differences in axon coverage from any site (anterior, middle or posterior) at any postnatal age, when total area of coverage was expressed as a percentage of total cortical area, regardless of the substrate of axon extension.

Tracer placements that happened to bridge more than one cortical area, as judged by retrograde labeling of both primary visual and somatosensory thalamic nuclei, might be expected to produce larger ranges of axon travel if each cortical area specifies a unique list of termination addresses, as contrasted with a model of initial axon outgrowth independent of cortical area identity. Though the number of cases we could use to address this question is small, examination of the area of cortex labeled by tracer injections in the several cases that resulted in retrograde label to both somatosensory and visual thalamic nuclei (45.0%, n = 3, across ages) compared to injections that labeled either one or the other class (70.2%, n = 7, across ages), however, showed the opposite, though non-significant trend.

Despite the general similarity of widespread coverage patterns from P1–P9, some local patterns were evident. As illustrated by the gradient outlines in Figure 5, long-range connections from anterior cortex extend asymmetrically posterior and laterally towards middle cortex, not colonizing far posterior cortex. Axons labeled by injections in middle cortical areas appear reciprocally focused on anterior cortex with few posterior projections. The majority of developing intracortical projections from posterior cortex is confined to the posterior cortex itself, although from all sites a number of axons typically extend to presumed medial limbic regions.

#### Axon extension and trajectory changes in the white matter

Axon travel in the conventionally recognized pathway, the white matter, is summarized in Figure 6. Travel in the white matter is more confined than travel in the overlying substrates, and more anisotropic, as shown by a comparison of the axon coverage in Figure 6 to the outlines of axon coverage in the collapsed views illustrated in Figure 5.

**Figure 7:**
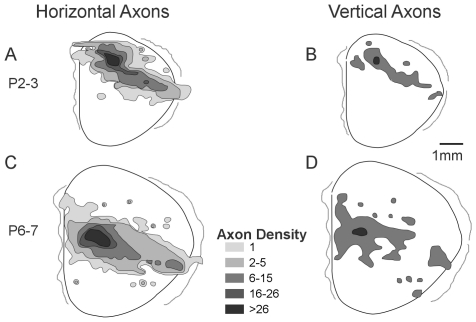
Vertically-turning axons. Comparative views of axon extension in cortex including gradients of only those axons extending horizontally and parallel to the white matter at ages P2–3 (A) and P6–7 (C) together with outlines of areas where vertically-oriented axons extending perpendicular to the white matter were found at each age (B and D). Outlines are representative of projections patterns found even at early ages in which labeled axons are found in areas both continuous and non-contiguous (possible target) with the injection site.

Because intracortical axons travel in large numbers through the cortex as well as the conventionally identified white matter, these schematized dorsal representations of axon populations do not distinguish axons traveling intracortically from those which exit the cortex, travel in white matter, and re-enter cortex to terminate. The generally uniform picture of axon extension that the dorsal view reconstructions suggest is perhaps at odds with the presence of a large number of abrupt trajectory shifts in axon tracks which might suggest alteration axon extension by detection of an areal boundary (e.g., Figure 4C, left). Trajectory shifts in white-matter axons might indicate regions of cell-to-substrate recognition that specify unique termination zones. For the two distributions which most strongly suggested the development of a particular termination focus, one early and one late in development, we charted every axon that turned from a horizontal trajectory to travel vertically (Figure 7). The locations of abrupt trajectory shifts populated the entire area of extension, however, producing a distribution that was simply a reduced form of the overall distribution. This pattern is more suggestive of random sampling of the substrate by the axon population prior to target selection [45], [46]. Overall these relatively uniform distributions resemble those seen in studies which reconstructed single-axon termination patterns within single areas in larger brains [25].

#### Synopsis of typical outgrowth pattern

Given the empirical observations above, we characterize the typical outgrowth pattern as having the following features: (i) having areal coverage larger than half of the cortical hemisphere; (ii) comprising axons travelling in both the white and gray matter which traverse comparable distances; (iii) having a footprint with greater extent along the ML axis than along the AP axis, with travel in the white matter being comparatively more constrained in this regard; (iv) being largely independent of the location of its source. With these features in mind, we developed the following framework to arrive at a quantitative description.

#### Modeling outgrowth distributions

**Figure 8:**
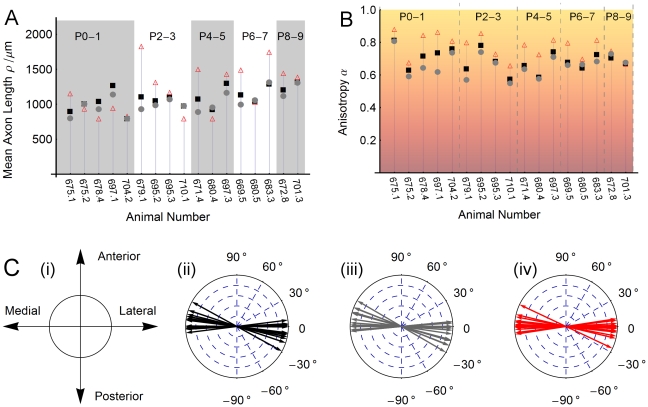
Fitting axon distributions. (**A**) **The fitted mean length of an axon** in the collapsed (black squares), cortex (gray dots) and WM (red triangles) outgrowth distributions for each animal (assuming a gamma distribution with shape parameter equal to 2). (**B**) **Measured anisotropy** in the collapsed (black squares), cortex (gray dots) and WM (red triangles) outgrowth distributions for each animal. We note that white matter axons travel more anisotropically than those traversing gray matter. See Figure 1 and the Methods section for details of our approach to quantifying anisotropy in the measured distributions. (**C**) **Preferred direction of travel.** (**i**) Orientation of our coordinate system relative to the anterior/posterior and medial/lateral axes of the cortical hemisphere. The preferred axis of axon travel, shown here for each animal's (**ii**) collapsed, (**iii**) cortex and (**iv**) white matter distributions tends to align closely with the ML axis.

Probability densities for the angular and radial components, denoted 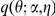 and 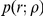, respectively, were fitted to the observed outgrowth distributions. The resulting values of the characteristic length 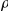, anisotropy 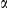 and tilt 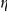 for each dataset are shown in Figure 8, and can be summarized as follows.

The radial distribution shows a marked departure from uniformity (see Figure 8B). The measured values of anisotropy 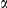 for collapsed distribution have mean 0.69 and standard deviation 0.07 on our 0 to 1 scale (see Figure S2 for fits to data). There is a clear tendency for white matter distributions to be more anisotropic (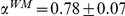) than distributions measured only in the cortex (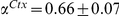). The tilt 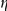 is such that the preferred direction of travel is almost collinear with the ML axis, as illustrated in Figure 8C (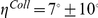).

The length distribution of axons in the white matter, gray matter and collapsed distributions were well fit by gamma distributions (see Figure S1 for fits to data), with mean lengths as shown in Figure 8A. There is a tendency for distributions measured in older animals to be longer and for white matter axons to travel greater distances. Taking the ratio 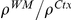 for each animal, we find that 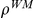 is on average 119±33% as long as 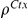 - indicating a trend of axons travelling in white matter reaching further than those traversing cortex only. This greater length is not incompatible with the lesser areal coverage of white matter axon distributions; travel in this compartment was also noted to be more anisotropic and therefore has a more slender footprint.

#### Spatial network modeling

We sought to create a model of the early cortical network which was faithful both to the qualitative characteristics and the measured distributions of axon outgrowth. We also wanted the nodes of this network to be biologically meaningful but representing neurons as individual nodes would have made our model computationally unwieldy. We instead take our nodes to be cortical populations or units, comprising all the neuronal cell bodies and local processes within a small volume of the cortical sheet, much like the “cortical output units” of Innocenti and Vercelli [47]. We define these units as 100 µm×100 µm squares on a 2-dimensioal cortical sheet, so that they have roughly the dimensions of a dendritic arbor. Hence our “local” length-scale is set at ∼100 µm. The number of nodes is thus conducive to constructing and analyzing our simulated networks on a desktop computer.

The simulated networks are found to have the small world property but are not scale free. The small world index is measured to be 4.57±0.17, indicating our networks possess short path lengths comparable with those of random networks while also having far higher clustering. This may have been anticipated given the form of the axonal distributions employed, which have a preponderance of local connections and relatively few long links. The networks are not in the class of so-called scale-free networks, with neither their in-degree nor out-degree having the required power-law distributions. The out-degree is, by construction, the same for every node and is equal to the bespoke number of efferent axons per node, 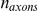. The in-degrees are in close agreement with those for a random network, only differing slightly from the Poisson distribution one would encounter in that case. We can see why such an in-degree distribution arises: if we ignore boundary effects, then nodes attract afferent axons with a probability proportional to the area of their respective Voronoi cells, the polygons in the plane enclosing all points to which a given node is the closest node. Given our method of distributing nodes in the plane, the preponderance of Voronoi cells not adjacent to borders will have area comparable with that of our 100 µm×100 µm grid squares. Such nodes therefore attract afferent edges with approximately equal probability, thus giving rise to the observed narrow, near-Poisson distribution of in-degrees.

#### Anisotropy of axonal distributions: consequences for efficiency, robustness and modularity

Anisotropy in axonal distributions may lead to a more volume-efficient wiring scheme but would seem, prima facie, to entail negative repercussions for the resulting network structure. Given that increased anisotropy in laying down axons leads to a smaller ensemble of possible networks (to see this consider the limiting case where axons are restricted to travel along only one axis), we were curious as to what advantages it might bestow. While investigating the effect of varying anisotropy *α* on the topology of simulated networks, we also sought to determine whether increased *α* might lead to a more compact packing of axons in space. We begin with the observation that an axon's length, and thus its volume, is increased if it must deviate to avoid another axon (see Figure 3). Therefore, encounters with other axons tend to increase the total volume requirement.

**Figure 9:**
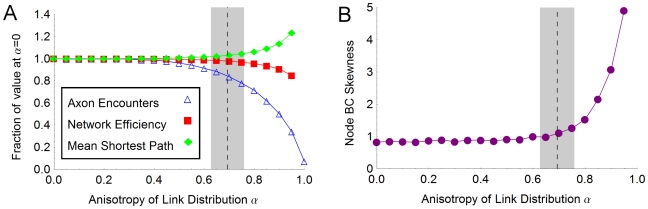
Effect of anisotropy. (**A**) **Packing volume and network paths.** In our simulations increased anisotropy *α* leads to a reduced number of axons encouters but also a decrease in network efficiency *E* and an increase in mean shortest path length *l*. The dashed line marks the mean empirical value of *α* for collapsed axon distributions and the extent of the shaded region indicates the standard deviation in that quantity. Each data point is the average result for 10 networks, each generated with *N*  =  2500 nodes, having *n_axons_  = * 10, drawn from under a distribution having anisotropy as indicated and length distributed as a gamma-2 distribution with average length 1000 µm. (**B**) **Node betweenness centrality.** Increased anisotropy *α* leads to an increased right-skewness in the distribution of node betweenness centrality in the generated networks. The dashed line and shaded region, respectively, denote the mean and standard deviation of the empirical values for *α*. These data were generated using the same network parameters as in (A). See also Figure S3 for a comparison of the histograms of the betweenness centrality distribution at *α  = * 0.02, *α  = * 0.7 and *α  = * 0.9.

Our spatial network model predicts that there exists a narrow range of values for the anisotropy parameter *α* which reduces the extra volume requirement of crossing axons without significantly impacting the ease of communication within the network, as measured by the efficiency *E* (see Figure 9A). Increasing the anisotropy beyond this range is predicted to reduce network efficiency. We find that the empirically measured values of anisotropy fall within this range for both white matter and gray matter travel.

**Figure 10:**
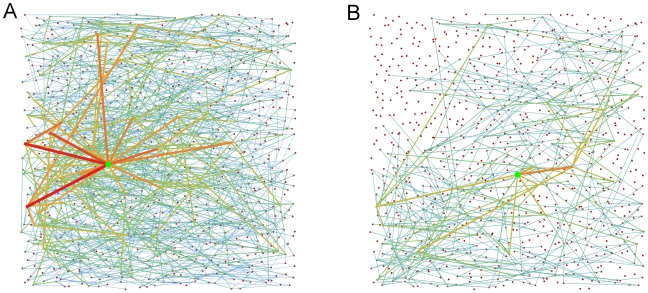
Contrasting nodes of high and low node betweenness centrality on the same network. (**A**) and (**B**) each show a different subset of the shortest paths on the same network. In (**A**), the node on the graph with the highest betweenness centrality is marked by the green dot. All the shortest paths which travel through that node are shown. Almost 1.5% of this directed graph's *N*(*N* - 1) paths pass through the node. The edges used by these paths have color and thickness reflecting the number of paths traversing that edge. By contrast, (**B**) shows a node of lower betweenness centrality which is on less than 0.1% of the network’s shortest paths. The network has *N*  =  900 nodes, *n_axons_  = * 5, average axon length 800 µm and *α  = * 0.65.

Increased anisotropy may lead to networks which are more vulnerable in the case of central nodes failing. The presence of such nodes is indicated by a more skewed distribution of node betweenness centrality (see Figure 9B; also Figure S3 for histograms of betweenness centrality distributions). The betweenness centrality *b_i_* of the node *i* quantifies the relative contribution of individual network elements to the collection of shortest paths on the network (see Figure 10). Conversely, it can be seen as a measure of how detrimental the removal of that element might be to the functioning of the network as a whole. We observe that increased anisotropy in the spatial distribution of links leads to an increased positive skew in the distribution of node betweenness centrality in simulated networks (Figure 9B). The right tail of the distribution becomes increasingly “heavy”, signifying the emergence of a small number of nodes with betweenness centrality much higher than the average value. As studies in other complex networks have shown [48], the existence of such highly central nodes renders the network as a whole more vulnerable to functional disruption in the event of their failure. It is also the case, however, that communication on such a network is more robust to the failure of a typical node, i.e. one which is not among the few highly central nodes.

The possibility of nodes becoming overloaded may be another reason to disfavor highly anisotropic wiring schemes. Assuming that information is propagated along the shortest paths between nodes, betweenness centrality can be interpreted as a measure of how much traffic a node handles. Given the finite neural populations comprising our nodes, they will have a limited capacity to process or propagate information. Hence, with highly anisotropic wiring, nodes which are very central may be at risk of becoming overloaded, thereby affecting the reliability of communication on the network.

**Figure 11:**
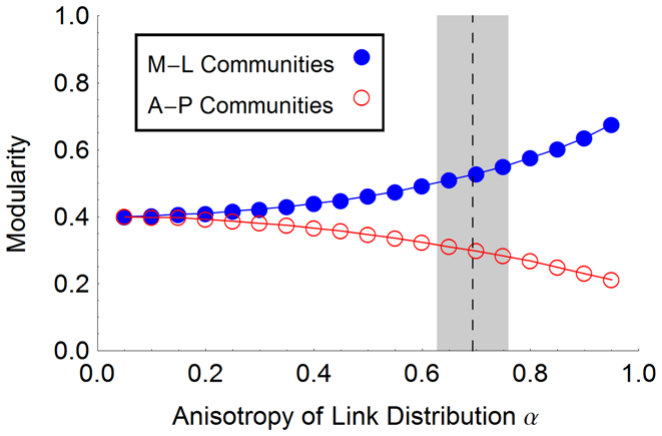
Modularity versus anisotropy of axon outgrowth. For the range of anisotropy values, we calculated the modularity of our networks with respect to two different community assignments. Assigning nodes to 4 approximately equal rectangular blocks, spanning the medial-lateral extent of our model cortex and having height equal to one fourth of the anterior-posterior extent, we see that modularity increases with anisotropy. However, using a partitioning which assigns nodes to communities extended along the anterior-posterior axis instead, we observe a decrease in modularity. In both cases, the networks are more modular than the comparable randomly wired network. This is because the prevalence of relatively short edges in our networks leads to clustering among nearby nodes in either set of communities.

Looking at the modularity of our networks as anisotropy is increased, we observe that partitioning the network into communities aligned with the medial-lateral axis of our grid is increasingly favored over choosing communities aligned perpendicular to that axis (see Figure 11). This may favor a layout in which more strongly connected and functionally related cortical areas have that same axial alignment. We investigated modularity with respect to partitioning the nodes into 4 spatially defined communities: first, communities which were (approximately) equally-sized rectangular blocks of nodes, aligned with the medial-lateral axis; second, a similar arrangement but with the rectangular blocks now parallel to the anterior-posterior axis. In both cases, the preponderance of short links, which leads to spatial clustering, ensured that our networks were more modular than the comparable network wired uniformly at random. However, the modularity was seen to increase significantly with anisotropy for the medial-lateral partitioning while decreasing for the anterior-posterior partitioning.

## Discussion

The neuroanatomical results we present in this paper show a surprising independence between the early patterns of axon outgrowth and the cortical region of their origin. In these small brains, axons extend to the limits of their growth through the cortex itself, and also in the white-matter tracts under the cortex. There is no obvious variation in axon “behavior” between axon populations arising from sites of origin near cortical boundaries, and axons traversing future borders of cortical areas. Axons originating from delimited regions cover a third to a half of the entire cortical area. The network model of cortical connectivity inferred from these outgrowth distributions suggests that at this scale the cortical network has a high small-world index and is not scale-free but rather has both in- and out-degrees which are narrowly distributed (“single-scale”). Our spatial model suggests that the unexpected anisotropy of initial axon outgrowth may represent a partial solution to the problem of minimizing the volume requirement of cortical connections while simultaneously maintaining network efficiency.

Reduced network efficiency has straightforward interpretations in the context of neural networks, where a primary goal is the cost-effective and timely exchange and integration of information. Increased average path lengths, too, can serve only to incur higher costs and error rates in propagating information on the cortical network. Further, the increasingly right-skewed distribution of node betweenness centrality with increased anisotropy in axon travel hints at yet another potential constraint: highly central nodes render the network liable to suffer a marked increase in path lengths in the event of their failure or overload. Clearly, some trade-off must exist between these deleterious effects and any benefits arising from reduced wiring volume. We do not know what relative weights one should assign to such costs and benefits in order to achieve the optimal trade-off. However, our results suggest that the axonal patterns recorded in this study may achieve such a balance. Further, there is evidence that different mammalian orders may have evolved disparate solutions to the problem of supporting and connecting increased numbers of neurons in the neo-cortex [49], [50]. Anisotropy of axonal outgrowth patterns may be just one aspect of the various strategies available to taxa in solving this problem.

We suggest that two features of the outgrowth patterns described here may contribute to the layout of the cortex: highly central nodes in this developing cortex may seed future hub regions and modularity may favor certain spatial layouts for cortical areas. Regarding the former, one possible effect of a skewed node centrality distribution is to distinguish potential future hubs in our network of cortical units. Although network hubs typically have higher-than-average degree, the degree statistics of our nodes are narrowly distributed and uniform across 2-dimensional space. This is in contrast with reports of broader degree distributions in the adult cortical network [51]. However, in our model networks, nodes with high betweenness centrality begin to appear as anisotropy is increased. Such nodes are uniformly distributed in space, but may provide seed sites for the emergence, in interaction with activity from sub-cortical projections, of structural network hubs, and for a broader distribution of node degrees [6], [24]. As development proceeds, elevated centrality may lead activity at such sites to be correlated with that at distal regions of the cortex, leading to increased persistence of afferent projections. Secondly, we observed that the modularity of our networks favors the formation of communities which extend parallel to the medial-lateral axis. The overall layout and separation of sensory and motor modalities in the cortex is established by the embryonic polarization of the cortical sheet [52]. That polarization may enable the emergent modularity of the cortical network we have modeled. Later in development, this modularity may itself guide the formation of features of cortical network structure.

In this study, we have only examined the connectivity structure “implied” by the pattern of axon outgrowth also making the assumption that an axon has exactly one arbor, occurring at the extent of its travel. We have neither demonstrated that synaptic connections have been made by these axons at the limits of their extents, nor that the connections are permanent. Axon tracing studies have demonstrated that a typical axon may branch once or more and may have arbors at sites other than its most distal arbor [53]. As to the effects of such limitations in our approach, we expect the measured anisotropy of outgrowth to be largely unchanged (assuming that branches of the same axon proceed independently). The length distributions we have fitted, however, will have under-represented axon ramifications at shorter lengths. The effect of including more ramifications at shorter distances from the neuron's cell body would be to further increase the modularity and clustering (and hence, we expect, also the small-world index) of our networks.

Our network model presents a reduced representation of the cortical network by taking as its nodes “cortical units” rather than the greatly more numerous constituent neurons – an approach consistent with the notion, presented in several studies, that it is appropriate to consider computational units having the scale of, for example, ocular dominance columns [47]. Implicit in our model, however, is the further assumption that cortical connectivity at this intermediate scale can also be usefully depicted using a representative sample of “edges” far smaller in number than the axonal connections they hope to mimic. The cross-comparability of neural networks sampled at different scales and resolutions is a topic of current interest in the neuro-imaging community [54].

We note that travel in the white matter as measured in this study is only slightly longer than the cortex-only travel. For this reason, our network model contained only one class of links, modeling all axons as identical. From the empirical data it is clear that axons traversing the gray matter alone can span the cortex. However, while the axons travelling in the white matter are not very much longer than their gray matter counterparts, they do represent a significant portion of the total axon population. This fact suggests these axons, with their capacity for faster and more reliable spike propagation, bestow some functional advantage even in a small brain. Further, this length mismatch could suggest that the size of the hamster cortex lies near an upper limit for what can be spanned by gray matter axons alone.

Our model is easily extended to accommodate two (or more) classes of axon. One could, for example, employ a short class, to mimic unmyelinated axons having a reach less than the dimensions of the cortex, and a longer class, representing connections having a length-scale comparable with the extent of the cortex. In this manner our model may elucidate the empirically observed scaling of white matter volume with increased cortical size [55]. This will necessitate deciding on some minimum threshold for network performance (in terms of efficiency, shortest path lengths, or similar), and then finding the possible distributions and number of white matter connections required to achieve that standard.

In conclusion, our goal was to succinctly capture the salient features of the empirically-measured initial outgrowth distributions in a simple model with a minimal number of parameters. Such a concise model, having a small parameter space, is well suited to exploring the developmental implications of changes in these parameters. Ultimately, we would like to test our model's predictions for connectivity in larger cortices, thereby gaining insight into how developmental programs have evolved to achieve efficient communication in larger mammalian brains.

## Supporting Information

Figure S1
**Fits to data for the length distribution function**. Shown here in blue is the empirically measured function 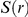 and, in red, our model, 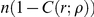, where 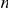 is a normalizing constant and 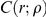 is the cumulative distribution function of the gamma(2) distribution, 
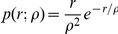
.(JPG)

Figure S2
**Fits to data for the angular distribution function.** Shown here in blue is the empirical function 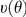 and, in red, the fitted angular probability distribution 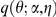 for the collapsed axon distributions. The parameter 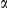 (“anisotropy”) determines the height of both peaks and 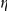 (“tilt”) determines the location on the circle of the diametrically opposed peaks.(JPG)

Figure S3
**Histograms of node betweenness centrality for several values of values of anisotropy** 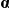 **.** The right tail of the distributions of betweenness centrality values is seen to become heavier with increased anisotropy 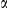 of the axon distribution. The inset provides an enlarged view of the area demarked by the dashed rectangle.(JPG)
