# Supplementary figures and images for: Network Structure Implied by Initial Axon Outgrowth in Rodent Cortex: Empirical Measurement and Models
Source: PLoS One. 2011 Jan 11;6(1):e16113. doi: 10.1371/journal.pone.0016113 (PMC3019165; doi:10.1371/journal.pone.0016113)

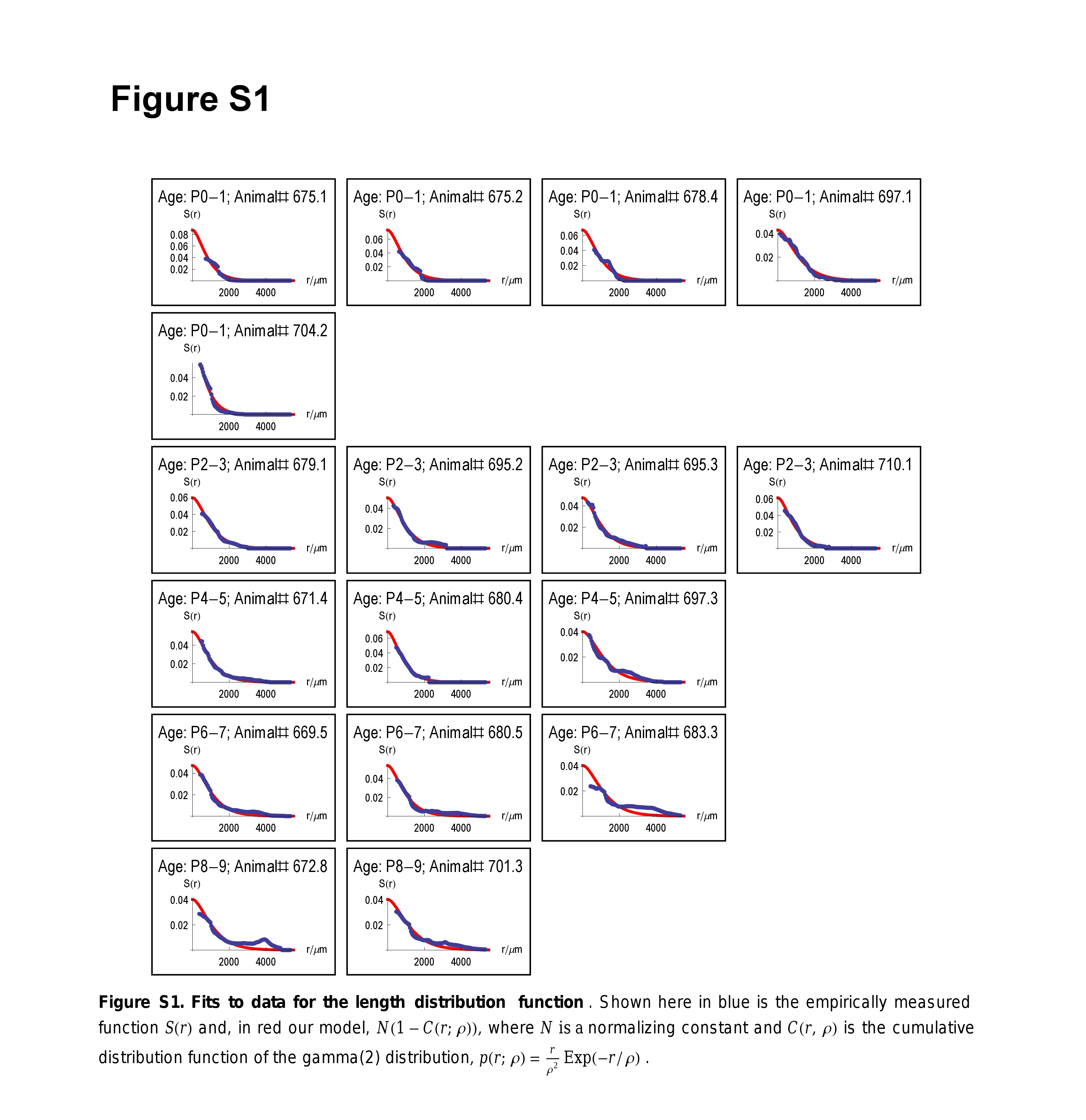

Supplement: Figure S1 — Fits to data for the length distribution function. Shown here in blue is the empirically measured function and, in red, our model, , where is a normalizing constant and is the cumulative distribution function of the gamma(2) distribution, . (JPG) [file pone.0016113.s001.jpg]

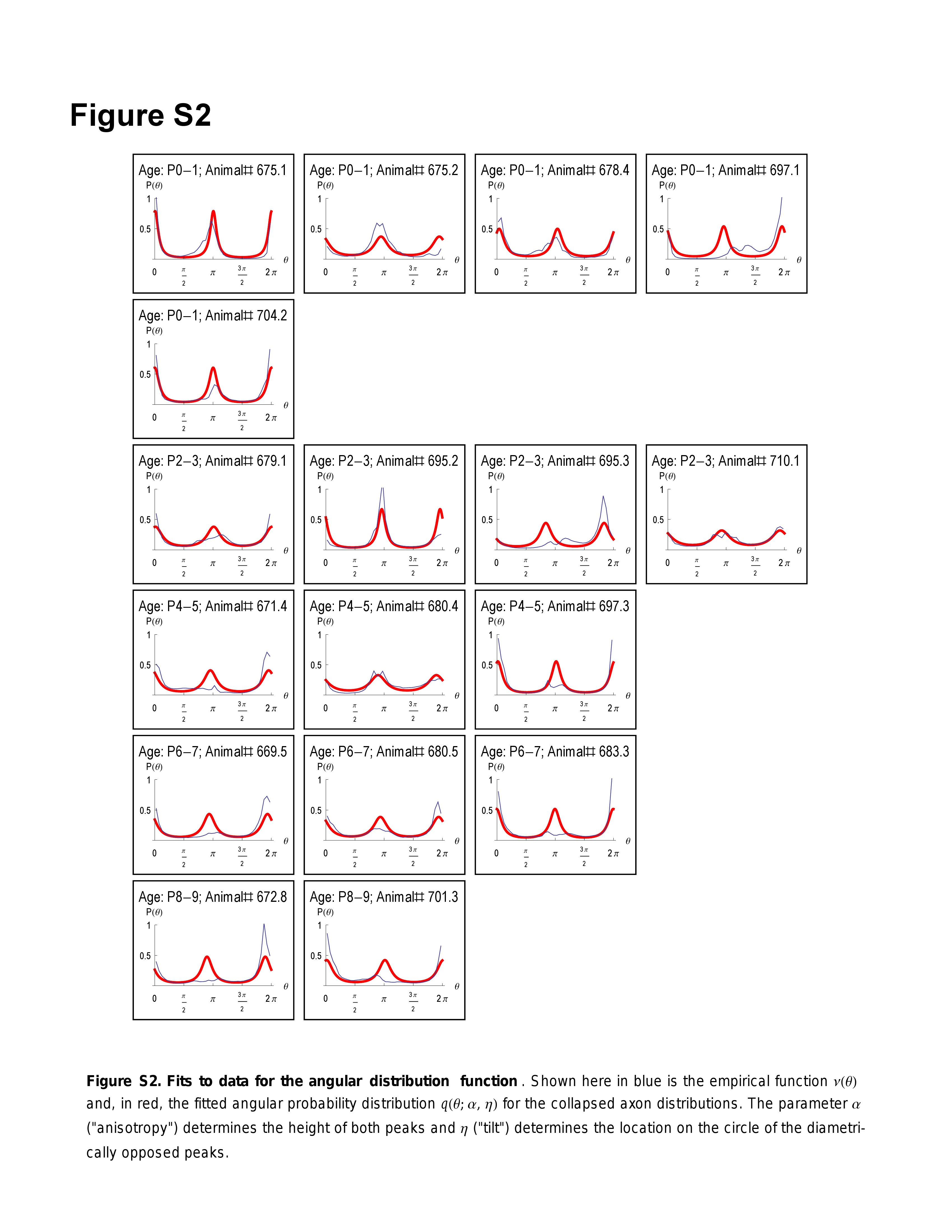

Supplement: Figure S2 — Fits to data for the angular distribution function. Shown here in blue is the empirical function and, in red, the fitted angular probability distribution for the collapsed axon distributions. The parameter (“anisotropy”) determines the height of both peaks and (“tilt”) determines the location on the circle of the diametrically opposed peaks. (JPG) [file pone.0016113.s002.jpg]

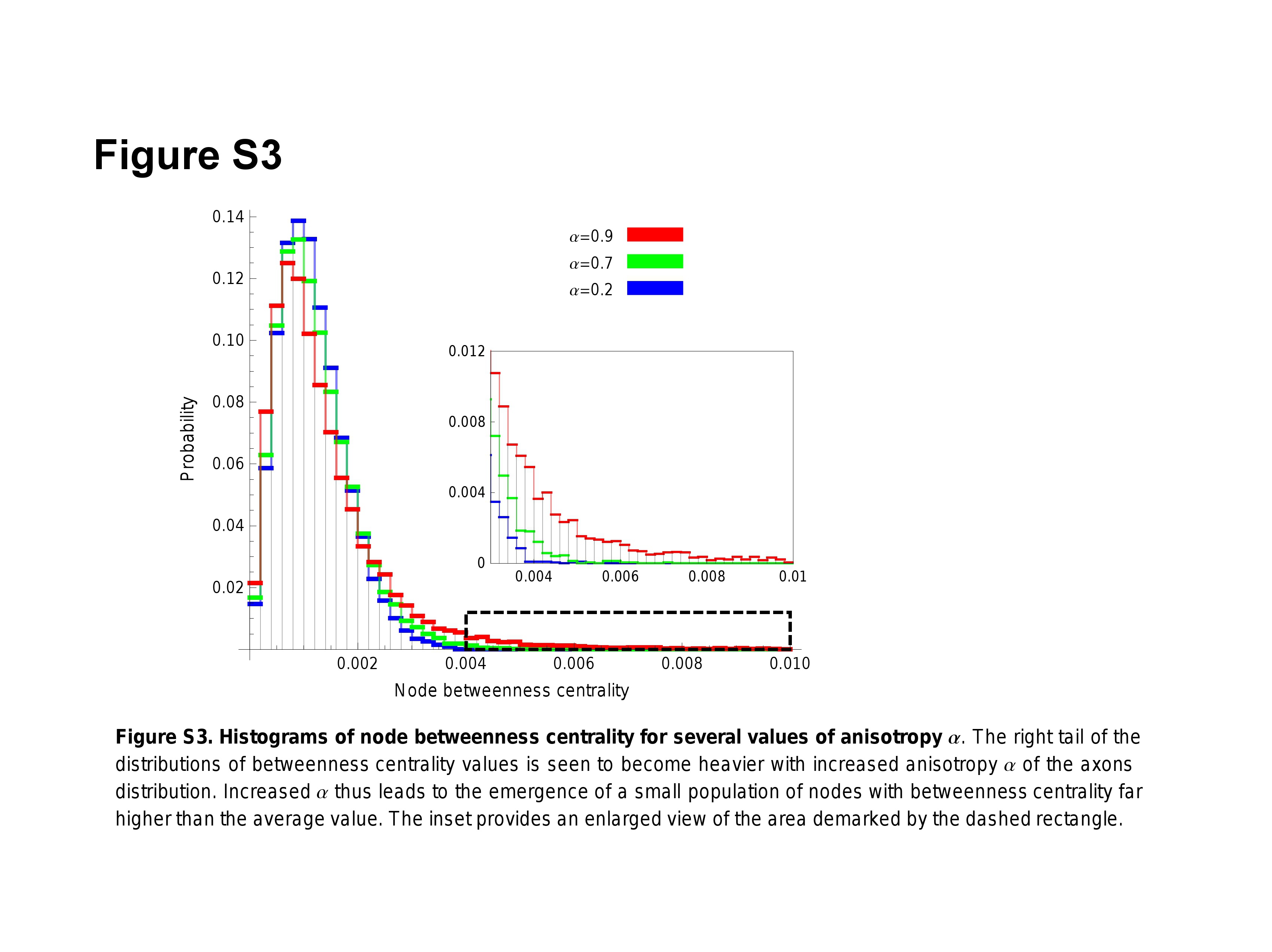

Supplement: Figure S3 — Histograms of node betweenness centrality for several values of values of anisotropy . The right tail of the distributions of betweenness centrality values is seen to become heavier with increased anisotropy of the axon distribution. The inset provides an enlarged view of the area demarked by the dashed rectangle. (JPG) [file pone.0016113.s003.jpg]
